# Recapitulation of dyssynchrony-associated contractile impairment in asymmetrically paced engineered heart tissue

**DOI:** 10.1016/j.yjmcc.2021.10.001

**Published:** 2022-02

**Authors:** Justus Stenzig, Marc D. Lemoine, Aaltje M.S. Stoter, Kinga M. Wrona, Marta Lemme, Wesam Mulla, Yoram Etzion, Thomas Eschenhagen, Marc N. Hirt

**Affiliations:** a Department of Experimental Pharmacology and Toxicology, University Medical Center Hamburg-Eppendorf, Hamburg, Germany; b DZHK (German Centre for Cardiovascular Research), Partner Site Hamburg/Kiel/Lübeck, Germany; c University Heart and Vascular Center, University Medical Center Hamburg-Eppendorf, Hamburg, Germany; d Department of Physiology and Cell Biology, Regenerative Medicine, Stem Cell Research Center, Ben-Gurion University of the Negev, Beer-Sheva, Israel

**Keywords:** Cardiac dyssynchrony, Tissue engineering, Engineered heart tissue, Pacing, Resynchronization, AE, Afterload enhancement or afterload enhanced, CRT, Cardiac resynchronization therapy, DHF, Dyssynchrony associated heart failure, EHT, Engineered heart tissue, fps, Frames per second, hEHT, Human engineered heart tissue, HF, Heart failure, HiPSC, Human induced pluripotent stem cell, LBBB, Left bundle branch block, LV, Left ventricular, MOI, Multiplicity of infection, rEHT, Rat engineered heart tissue, RV, Right ventricular

## Abstract

**Background:**

One third of heart failure patients exhibit dyssynchronized electromechanical activity of the heart (evidenced by a broad QRS-complex). Cardiac resynchronization therapy (CRT) in the form of biventricular pacing improves cardiac output and clinical outcome of responding patients. Technically demanding and laborious large animal models have been developed to better predict responders of CRT and to investigate molecular mechanisms of dyssynchrony and CRT. The aim of this study was to establish a first humanized *in vitro* model of dyssynchrony and CRT.

**Methods:**

Cardiomyocytes were differentiated from human induced pluripotent stem cells and cast into a fibrin matrix to produce engineered heart tissue (EHT). EHTs were either field stimulated in their entirety (symmetrically) or excited locally from one end (asymmetrically) or they were allowed to beat spontaneously.

**Results:**

Asymmetrical pacing led to a depolarization wave from one end to the other end, which was visualized in human EHT transduced with a fast genetic Ca^2+^-sensor (GCaMP6f) arguing for dyssynchronous excitation. Symmetrical pacing in contrast led to an instantaneous (synchronized) Ca^2+^-signal throughout the EHT. To investigate acute and long-term functional effects, spontaneously beating human EHTs (0.5–0.8 Hz) were divided into a non-paced control group, a symmetrically and an asymmetrically paced group, each stimulated at 1 Hz. Symmetrical pacing was clearly superior to asymmetrical pacing or no pacing regarding contractile force both acutely and even more pronounced after weeks of continuous stimulation. Contractile dysfunction that can be evoked by an increased afterload was aggravated in the asymmetrically paced group. Consistent with reports from paced dogs, p38MAPK and CaMKII-abundance was higher under asymmetrical than under symmetrical pacing while pAKT was considerably lower.

**Conclusions:**

This model allows for long-term pacing experiments mimicking electrical dyssynchrony *vs.* synchrony *in vitro*. Combined with force measurement and afterload stimulus manipulation, it provides a robust new tool to gain insight into the biology of dyssynchrony and CRT.

## Highlights

• Cardiac electrical dyssynchrony can be modeled *in vitro* in engineered heart tissue.
• Asymmetrically paced engineered heart tissue recapitulates important protein markers of cardiac dyssynchrony.
• Asymmetrical *vs.* symmetrical pacing *in vitro* allows for studying synchrony in cardiomyocytes in an isolated manner.

## Graphical abstract

**Figure:**
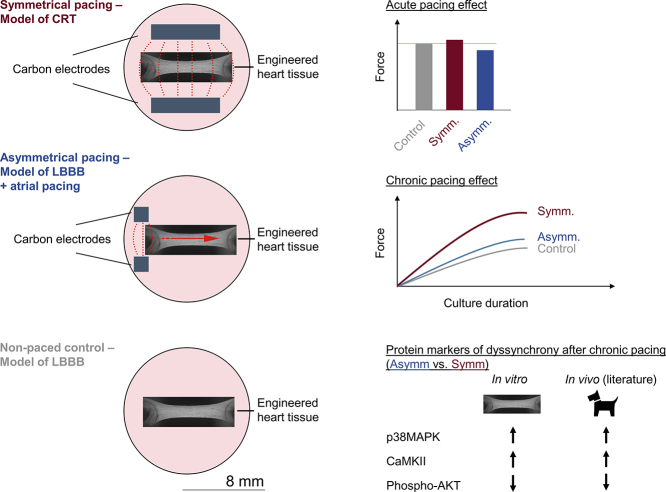

## 1 Introduction

The prevalence of heart failure (HF) is rising worldwide. Main reasons are aging of the population and the improved treatment of hypertension, valvular disease and coronary disease, which allows patients to survive these conditions longer, though often at the expense of a later development of HF [1]. About one third of heart failure patients exhibit electrical dyssynchrony (evidenced by a QRS-complex >120 ms in the surface ECG), which leads to ventricular mechanical dyssynchrony and worsens clinical outcome [2]. Iatrogenic right ventricular (RV) pacing can also induce dyssynchrony and impair left ventricular (LV) mechanics in a similar manner. Cardiac resynchronization therapy (CRT) for which a left ventricular pacing lead is added to approach simultaneous biventricular (BIV) pacing, is a potent modality to treat HF patients with dyssynchronous LV contraction [3,4]. A fraction of patients subjected to CRT (“responders”) exhibits an immediate increase in cardiac output, improved long-term LV function, reversal of cardiac remodeling and decreased mortality [5,6]. However, critical remaining issues include correct patient selection for CRT (besides QRS duration and a clear left bundle branch block) [7] and prediction of clinical outcome in non-HF patients exposed to dyssynchrony [3].

Recent data support the notion that molecular aspects are critical in determining cardiac response to dyssynchrony [8], but the exact details and the molecular mechanisms of the beneficial effects of CRT remain elusive. Current experimental models of dyssynchrony and CRT have limitations: they rely on large animals and are technically demanding, and thus are not available to most researchers. Using a novel methodology for efficient implantation of cardiac electrodes in rodents, it has been recently demonstrated that cardiac pacing of rodents mimics important electromechanical features observed in humans and may be used to study the biology of dyssynchrony and CRT [[9], [10], [11]]. Nevertheless, the complicated nature of *in vivo* studies makes it hard to obtain comprehensive insight into the mechanisms of dyssynchrony.

In this study, we followed the idea to model synchronous and dyssynchronous pacing modes *in vitro*, employing strips of engineered heart tissue (EHT). Several of their properties argue in favor of EHTs: they can be created from human cardiomyocytes (differentiated from human induced pluripotent stem cells (hiPSCs)) avoiding interspecies translational problems; they are accessible to numerous interventions and functional and molecular analyses and they have shown marked beneficial effects of prolonged uniform field stimulation (the equivalent of synchronous stimulation) in an earlier study [12]. Thus, we established EHT as a novel *in vitro* model to study molecular consequences and drug responsibility of dyssynchrony with increased throughput.

## 2 Methods

### 2.1 Generation, culture and analysis of rat engineered heart tissue (rEHT)

The workflow to generate rEHT has been described in detail previously [12,13]. All animal work has been reviewed and approved by the regional ethics review board of the Medical Council of Hamburg, Germany (approval number ORG516) and was conducted in accordance with the Guide for the Care and Use of Laboratory Animals as adopted by the United States National Institutes of Health (8th edition, revised 2011). In brief, hearts of neonatal rats (postnatal day 0 to 3) were excised and ventricular heart cells (all cell types, cardiomyocyte content ≈60%) [14] were isolated by a trypsin/DNase digestion. Per EHT 500,000 cells were mixed with fibrinogen (Sigma-Aldrich F8630) and thrombin (Sigma-Aldrich T7513) at a final volume of 100 μl and then quickly pipetted into agarose casting molds, into which two hollow silicone posts protruded from a silicone EHT rack placed above the casting molds. After 90 min, the fibrin block containing the heart cells could be taken out of the molds with the help of the two silicone posts, in between which the fibrin strip was spanned. Next, these EHTs were transferred to 24-well culture dishes filled with medium consisting of low glucose (1 g/l) DMEM (Biochrom F0415), 10% heat inactivated horse serum (Gibco 26050), penicillin/streptomycin (each 100 U/ml, Gibco 15140), insulin (10 μg/ml, Sigma-Aldrich I9278) and aprotinin (33 μg/ml, Sigma-Aldrich A1153, to prevent rapid fibrin degradation). Medium and pacing unit changes were synchronized and scheduled three times a week. All cell culture incubators were set to 100% humidity, 37 °C, 7% CO_2_ and 40% O_2_.

### 2.2 Generation, culture and analysis of human engineered heart tissue (hEHT)

For hiPSC experiments an in-house control cell line derived from a healthy female donor, who provided written informed consent, was utilized. This has been approved by the regional ethics review board of the Medical Council of Hamburg, Germany (approval number PV4798). Microbiological sterility, genetic stability, pluripotency and cardiac differentiation efficiency were verified. The information is accessible at hpsreg.eu, the cell line authentication is UKEi003-C (previously ERC018sv1583). HiPSCs were first expanded and then differentiated into human cardiomyocytes in suspension culture. This multi-step protocol (embryoid body formation at day 0, mesoderm induction at day 1, cardiac differentiation starting from day 4) has been published earlier [15]. At the end of the differentiation (day 14–17), embryoid bodies were dissociated with collagenase II (Worthington, #LS004176) and an aliquot of the resulting cell population was analyzed by flow cytometry. For cardiac troponin T staining, 400,000 cells each were fixed and stained with a fluorescently labelled antibody directed against troponin T (Miltenyi #130-106-688), or a corresponding isotype control (Miltenyi #130-104-613). For SSEA-3 staining, cells were stained with an antibody directed against SSEA-3 (Becton-Dickinson #560879) and a fluorescently labelled secondary antibody and fixed subsequently. Here, secondary antibody only was used as control. A typical cellular composition is shown in Supplemental Fig. S1 with ≈93% cardiomyocytes (troponin T^+^) cells and as low as ≈10% undifferentiated cells (SSEA3^+^). Only differentiation runs with >80% troponin T positive cells were used for hEHT generation. Freshly dissociated cardiomyocytes were used for hEHT generation. Apart from using double the number of cells (1000,000 viable cardiomyocytes) and the addition of the anti-apoptotic agent Y-27632, all EHT generation steps were identical to rEHTs [15]. Medium and pacing unit changes were also synchronized but performed daily. The only difference to the culture medium used for rEHT was the addition of 300 nM ivabradine in hEHT culture (Sigma-Aldrich #SML0281) to reduce spontaneous frequency.

Human EHTs were cultured on two different types of racks which only differed in the silicone stiffness. A very soft version with a stiffness of k = 0.28 mN/mm led to rather low final contractile forces, while a stiffer version with k = 0.80 mN/mm led to high force EHTs. For some experiments high force EHTs were heavily mechanically challenged during the last week of culture. This procedure is called afterload enhancement (AE) and has been previously described in detail [13]. In brief, the two silicone posts representing the anchoring points and providing the elastic resistance for each attached EHT were stiffened by metal braces. This procedure increased the afterload of EHTs by a factor of 14 (from k = 0.80 mN/mm to 11.5 mN/mm) and led to contractile dysfunction.

### 2.3 Construction and operation of pacing units

**Fig. 1:**
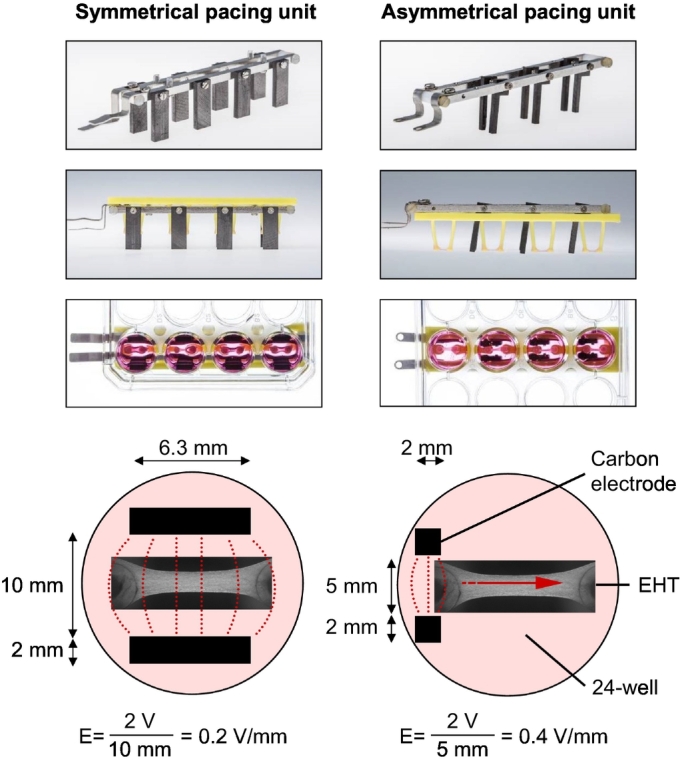
Pacing setup. Photographs of the symmetrical and asymmetrical pacing units, the same units with fitted engineered heart tissues (EHTs) and readily assembled in the cell culture dish filled with medium (from top to bottom). Schematic overview with exact dimensions and estimated electrical field strengths below.

Pacing units were designed to fit into standard 24-well cell culture dishes and to stimulate up to 4 EHTs per unit. Two different pacing strategies were pursued: symmetrical pacing and asymmetrical pacing. The pacing units for symmetrical pacing (Fig. 1, left) have been developed in the frame of an earlier project [12]. For asymmetrical pacing units, different geometries were tested. Particularly, the width of the carbon electrodes was reduced, the distance in between a pair of electrodes was diminished and the electrodes were positioned at one end of each EHT. The most extreme configuration, which could stimulate up to 3 EHTs per pacing unit, (Fig. 1, right) was used for all further experiments and is referred to as asymmetrical pacing unit. For all pacing units two stainless steel square bars (austenitic grade EN 1.4301, UNS S30400; Koch + Krupitzer, Schenefeld, Germany) served as conducting material and as scaffold for the carbon electrodes (#CG 1290, Carbon Graphite Consulting Klein, Siegen, Germany). The bars were isolated from each other by polyamide plastic screws (GHW, Niederkrüchten, Germany). Each pair of electrodes in a pacing unit and additionally all pacing units (when applicable) were connected in parallel, so the voltage set at the stimulator was equal to the voltage between each pair of electrodes. Unless otherwise described, voltage was set to 2 V and symmetric biphasic pulses of short duration (1.2 ms overall, 600 μs per polarity) were applied. The stimulation frequency for hEHT was 1 Hz, slightly higher than the spontaneous beating frequency under ivabradine treatment. For rEHTs an overstimulation would have required frequencies higher than 5 Hz during burst-pattern activity and could not be performed for longer periods. Thus, rEHTs were stimulated at 0.5 Hz, pacing only during the resting periods, which still had a strong effect in earlier studies [12]. The parallel arrangement resulted in high current demands (100–150 mA per pacing unit). Two types of high-current stimulators were used: Grass S88X Dual Output Square Stimulator (Natus Neurology Incorporated, Warwick, USA) and the C TYPE 224 stimulator (Hugo Sachs/Harvard Apparatus, March-Hugstetten, Germany). The latter one was customized to produce up to 800 mA output current.

Continuous electrical stimulation for all experiments started at day 4 of culture. The contractile parameters of EHTs were regularly determined in an automated contractility measurement system (EHT Technologies GmbH, Hamburg, Germany). Its central parts were an incubator with a glass roof and a camera mounted above it. The position of the camera could be controlled by software which also analyzed the recordings. Essentially, the rhythmic deflections of the silicone posts by the EHTs were recorded and were translated into contractility parameters, *e.g.*, contractile force using pattern recognition [16]. In all experiments, EHTs that were allowed to beat spontaneously but otherwise underwent all procedures as the paced groups served as controls. In contrast, the paced groups were also paced during the contractile measurements. Pacing units were reconditioned by washing them at least 3 times in distilled water for 12 h each before autoclaving.

### 2.4 Optical (fluorescent Ca^2+^-sensor) and electrophysiological (sharp microelectrode) analysis of electrical conduction in EHTs

The excitation (or more precisely the Ca^2+^-wavefront) propagating along the EHT was visualized with the fluorescent fast genetic Ca^2+^-sensor GCaMP6f. To this end, hEHTs were transduced at the time of casting with an adeno-associated virus, serotype 6 encoding for Fast-GCaMP6f-RS09 (Addgene plasmid #67160) under the control of a cytomegalovirus promoter at a multiplicity of infection (MOI) of 7 × 10^5^ [17]. The fluorescent signal was recorded with a Hamamatsu ImagEMX-2 fluorescence camera at a spatial resolution of 400 × 32 pixels (corresponding to 8.75 mm × 0.7 mm, *i.e.*, the approximate dimensions of an EHT) and a temporal resolution of 614 frames per second.

### 2.5 SDS-PAGE/Western blot analysis

For protein analyses at the end of the culture period, EHTs were removed from the silicone posts and frozen in liquid nitrogen. For some experiments, asymmetrically paced EHTs were cut into halves (paced and non-paced end). 100 μl extraction buffer (50 μl for split EHTs) composed of M-PER (Mammalian Protein Extraction Reagent, Thermo-Fisher #78501), Mini-Complete protease inhibitor (Roche #11837580001) and Phos-Stop (Roche #04906845001) was added, followed by a mechanical disruption (Qiagen Tissue Lyser, 2 × 30 s at 30 Hz), while all steps were performed on ice. Under reducing conditions (DTT containing Laemmli buffer) denatured proteins (of 75,000 cells and an according amount of fibrin matrix per lane) were separated by sodium dodecyl sulfate-polyacrylamide gel electrophoresis (SDS-PAGE) and blotted onto nitrocellulose membranes which were stained with Ponceau S to confirm equal protein loading per lane. After blocking in bovine serum albumin (5% in tris-buffered saline plus 0.1% Tween 20 [TBST]), membranes were washed (3 times, 5 min, TBST) and incubated with primary antibodies at 4 °C overnight. The following primary antibodies were used: anti-α-actinin monoclonal antibody (1:5000; Sigma-Aldrich #A7811); anti-CaMKII monoclonal antibody (1:1000; BD Transduction Laboratories #611292); anti-pCaMKII (Thr286) monoclonal antibody (1:1000; Cell Signaling #12716); anti-p38MAPK polyclonal antibody (1:1000; Cell Signaling #9212); anti-pAKT (Ser473) monoclonal antibody (1:400; Cell Signaling #9271); anti-cleaved caspase-3 monoclonal antibody (1:500; Cell Signaling #9664); anti-GAPDH monoclonal antibody (1:5000; HyTest #5G4). Following 3 washing steps (10 min, TBST), membranes were incubated with the respective horseradish peroxidase-conjugated secondary antibodies for one hour at room temperature. After final washing the immunoreactive bands were visualized by ECL detection (Pierce #32106). All original, uncropped blots can be found in the Supplemental Material (Supplemental Fig. S8).

### 2.6 Immunohistochemistry

EHTs were fixed for 24 h in a phosphate-buffered solution containing 4% formaldehyde stabilized with methanol. After embedding in paraffin, 3 μm sections were cut longitudinally in the median plane. Following an antigen retrieval with citrate-buffer (30 min, pH 6.0), staining was performed with the anti-myosin light chain 2, ventricular isoform (MLC-2v) monoclonal antibody (Synaptic Systems #310111, dilution 1:2000) and visualized with the multimer-technology based UltraView Universal DAB Detection Kit (Roche #760-500).

### 2.7 Statistics

Results are presented as mean ± SEM. All graphs and statistical tests (including linear regression and non-linear regression analyses) were created or performed in GraphPad Prism. One-way (repeated where applicable) ANOVA and Sidak's multiple comparison post-test were used for comparison of more than two groups. For results affected by two factors (*e.g.*, pacing mode and culture duration), two-way (repeated where applicable) ANOVA and a Sidak's correction for multiple comparisons was performed. When data were missing for repeated measurements a mixed model (REML = restricted maximum likelihood) was fitted and Sidak's multiple comparison tests were performed subsequently. Adjusted *p* *<* 0.05 or less was considered statistically significant. *P*-values are displayed graphically as follows: *p < 0.05, **p < 0.01, ***p < 0.001, ns = not significant.

## 3 Results

### 3.1 Asymmetrical pacing leads to dyssynchronous excitation of EHTs

The objective of this study – to model and investigate the consequences of synchronous and dyssynchronous pacing *in vitro* – required the construction of new pacing units. For synchronous pacing (corresponding clinically to normal physiology in the coordinatedly beating myocardium), we employed well characterized [12] symmetrical pacing units (Fig. 1, left) with large electrodes. They should create an almost homogenous electrical field (E ≈ 0.2 V/mm at 2 V) throughout the EHT. Different pacing units were tested for dyssynchronous pacing (corresponding clinically to right ventricular pacing and/or situations with broad QRS-complex). Due to the small distance between electrodes, the electrical field at the same voltage was presumably regionally stronger than for the symmetric setup, but centered at one end of the EHT. The voltage-duration relationship was assessed for the symmetrical and the asymmetrical setup, the latter with 5 and 10 mm distance between electrodes (Supplemental Fig. S2). Finally, a design was chosen with slim electrodes positioned at the minimum distance of 5 mm between each other and very close to one end of the EHT (Fig. 1, right).

**Fig. 2:**
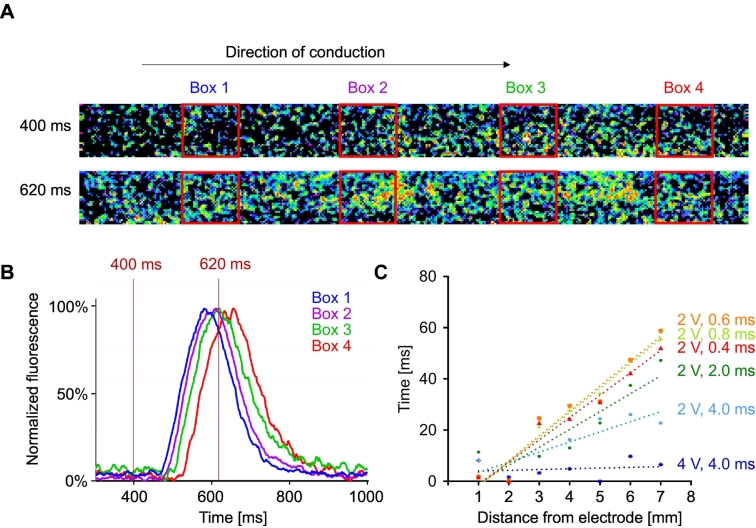
Electrical propagation. Visualization and analysis of the Ca^2+^-wave in human EHT (hEHT) for asymmetrical pacing under different settings. A Original recordings (400 × 32 pixel) with a Hamamatsu ImagEMX-2 fluorescence camera at 614 frames per second (fps). Red boxes (0.7 × 0.7 mm) represent the area for signal averaging at a non-excited (400 ms) and at an excited state (620 ms). Pacing electrodes were placed left of box 1. B Averaged fluorescence intensities in the four boxes plotted over time. C Temporal and spatial propagation of Ca^2+^-wavefront under different stimulator settings. (For interpretation of the references to colour in this figure legend, the reader is referred to the web version of this article.)

Before starting the long-term pacing experiments, we investigated whether the asymmetrical pacing truly triggered a depolarization wave from one end of the EHT to the other. Human EHTs were transduced with a fast genetic Ca^2+^-sensor (GCaMP6f) and the propagation of the fluorescent wave was recorded. Under appropriate stimulator settings, the fluorescent wave traveled along the long-axis of the EHT (Fig. 2 A). Spatial propagation of the Ca^2+^-wavefront in EHT was investigated (Fig. 2 B, for all settings see Supplemental Fig. S3) by high-speed fluorescence imaging. At a high voltage of 4 V or at a long duration of 4 ms and lower voltage, the Ca^2+^-signal appeared almost instantaneously throughout the EHT, arguing against asymmetrical pacing. However, with low voltage (2 V) and short pulse durations <1 ms per polarity the signal traveled through the EHT in approx. 50 ms, which represents a conduction velocity of ≈ 0.16 m/s at an EHT length of 8 mm (Fig. 2 C). This is about one third of the conduction velocity of a healthy human heart [18] which can be likely attributed to a lower expression of Nav1.5-channels in EHTs and potentially minimal Ca^2+^ buffering by the sensor itself (Supplemental Fig. S4) [17,19].

Moreover, we investigated whether the calcium wave - which should translate into a contractile wave, starting at the paced end and moving to the non-paced end of the EHT - leads to dyssynchronous movements of the two posts carrying the EHT. However, as expected, both posts moved synchronously under both symmetrical (Supplemental Fig. S5 A) and asymmetrical pacing (Supplemental Fig. S5 B). The latter is likely explained by the low mass (inertia) of the silicone posts which renders the minimal time delay between the paced and the remote end unmeasurable in our system with a temporal resolution of 100 fps.

### 3.2 Dyssynchronous pacing is inferior to synchronous pacing regarding contractile force and kinetics in rat and human EHTs

The central assumption of the present project was that asymmetric pacing leads to electromechanical dyssynchrony and therefore that the contractile function of asymmetrically paced EHTs should be worse than that of symmetrically paced EHTs. This hypothesis was tested in acute pacing experiments in human EHTs and long-term pacing experiments in both rat and human EHTs. Long-term pacing started at day 4 of culture and lasted for a minimum of 16 days of culture. For all experiments the groups were control (non-paced), symmetrically paced (2 V, 0.6 ms) and asymmetrically paced (2 V, 0.6 ms).

**Fig. 3:**
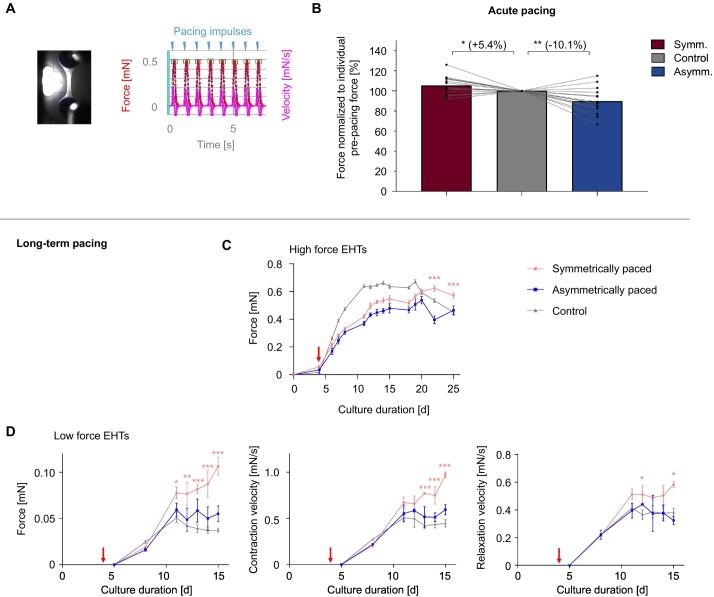
Acute and long-term effects of pacing on hEHT. A Left, still image of a video-recorded engineered heart tissue. View from above, white glow = illuminating LED. Right, excerpt from original recording of automatic visual tracing of EHT contractions, force over time derived from optical pattern recognition. B Acute effect of pacing on hEHTs. Control (never before paced) hEHTs were subjected to either symmetrical (*n* = 8) or asymmetrical pacing (*n* = 7). Forces (≈1 min after the onset of pacing) relative to each EHT's individual pre-pacing force. In a second round symmetrically paced EHTs were asymmetrically paced and *vice versa* leading to overall *n* = 15 per group. The order of pacing had no impact on the resulting forces. Parametric one sample tests compared to the control value of 100%. C Force of hEHTs with high forces (cultured on k = 0.80 mN/mm racks) and D Force, contraction and relaxation velocity of low force EHTs (cultured on k = 0.28 mN/mm racks). EHTs were either non-paced or continuously symmetrically or continuously asymmetrically paced from day 4 on (red arrow). For both pacing groups biphasic pulses at 1 Hz, 2 V and 0.6 ms duration per phase were applied. Asterisks indicate significant difference of the symmetrically paced group to the control group. Mixed model statistics followed by Sidak's correction for multiple testing. *N* = 9–33 EHTs per group in C, and *n* = 3–5 in D, *p < 0.05, **p < 0.01, ***p < 0.001. (For interpretation of the references to colour in this figure legend, the reader is referred to the web version of this article.)

The immediate effects of pacing were evaluated by recording forces (Fig. 3 A) of previously unpaced EHTs at their first contact with symmetrical or asymmetrical pacing. We could not identify mechanical dyssynchrony between the two posts. However, an increase of force (+5.4%) under symmetrical pacing and a decrease (−10.1%) under asymmetrical pacing could be observed (Fig. 3 B).

The first exploratory long-term pacing experimental series were performed in rEHTs. Rat EHTs beat in an uncommon burst (4 Hz) and resting phase-pattern, the two states typically alternating every 10–30 s. They were continuously stimulated at 0.5 Hz, which therefore only had an effect during the resting phases. The contractility results of the rEHTs (Supplemental Fig. S6 A–E) were partially inhomogeneous. There was a clear trend towards higher force in the symmetrically paced EHTs compared to their asymmetrical counterparts or the controls. After these pilot experiments we used hEHTs for all further experiments, which in contrast to rEHTs beat very regularly at frequencies around 0.5–0.8 Hz when cultured in the presence of 300 nM ivabradine. The frequency chosen for continuous pacing was 1 Hz. The results were more uniform and demonstrated moderate but significant superiority in high force EHTs at the end of the long-term pacing (Fig. 3 C) and marked superiority of symmetrical pacing regarding contractile force, velocity of contraction and velocity of relaxation in low force EHTs (Fig. 3 D). Here, after 11 days of pacing (at d15 of culture) forces of symmetrically paced EHTs were 2.86× higher (adj. *p* < 0.0001) than controls, while this effect was not observed for the asymmetrically paced EHTs (1.48× force of controls, adj. *p*-value = 0.15).

**Fig. 4:**
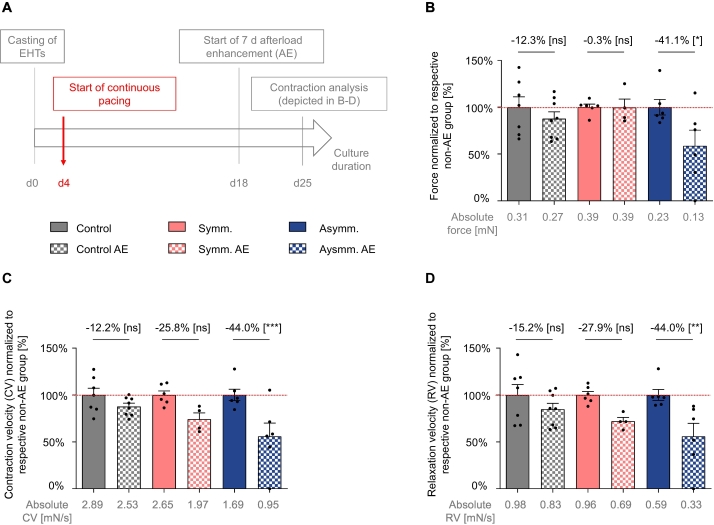
Afterload enhancement (AE). A Timeline of the experimental procedures. B Forces, C contraction and D relaxation velocities of control and long-term paced hEHTs (symmetrically or asymmetrically) after the AE-procedure (checked bars, 14-fold increased afterload/stiffness) normalized to EHTs without AE (filled bars). Pacing settings as in Fig. 3. Sample sizes *n* = 10–15 per pacing mode, and *n* = 4–7 per individual condition, two-way ANOVA followed by Sidak's correction for multiple testing, *p < 0.05.

EHTs of all three groups (control, symmetrically paced and asymmetrically paced) were then subjected to an AE procedure during the last week of culture (Fig. 4 A). We have previously shown [13] that this leads to a variety of changes characteristic of pathological cardiac hypertrophy, *e.g.,* contractile dysfunction. As mentioned above, asymmetrically paced EHTs had lower absolute forces than symmetrically paced EHTs, and the relative AE-effect on force (Fig. 4 B), contraction (Fig. 4 C) and relaxation velocity (Fig. 4 D) was most accentuated in the asymmetrically paced group.

### 3.3 Dyssynchronous pacing in human EHTs results in similar marker protein patterns as dyssynchronous tachypacing in dogs

**Fig. 5:**
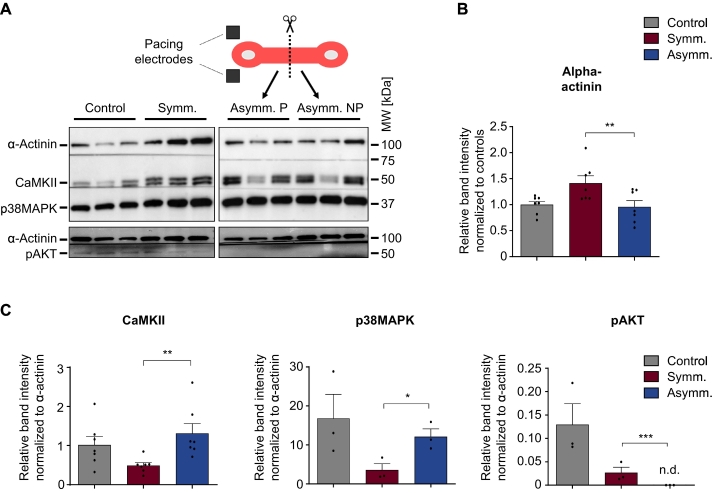
Protein markers of dyssynchrony. A Western blot of protein markers of dyssynchrony (CaMKII, p38MAPK, and phosphorylated AKT) or a sarcomeric protein (alpha-actinin) at the end of the long-term pacing procedure depicted in Fig. 3. For asymmetrical pacing, hEHTs were divided into two parts, paced (P) and non-paced (NP) end. B Quantification of Western blots for alpha-actinin. Dots in each group represent protein lysates from ≈75,000 input cells. C Quantification of Western blots for CaMKII, p38MAPK and phospho-AKT, all normalized to alpha-actinin values. One-way ANOVA followed by Sidak's correction for multiple testing and *n* = 3–7 in B–C, *p < 0.05, **p < 0.01, ***p < 0.001.

At the end of the long-term stimulation, human EHTs were analyzed for proteins that have been previously identified to be differently expressed or phosphorylated *in vivo*, namely the synchronized *vs.* dyssynchronized LV in dogs [20]. In this regard, the heart failure provoked in dogs by tachypacing in conjunction with left bundle branch block (LBBB) would correspond to the electrical state of EHTs at baseline, symmetrical pacing would correspond to CRT and asymmetrical pacing would correspond to continued atrial pacing in the presence of LBBB. Thus, in analogy to the literature [20], we initially analyzed the paced and the non-paced end of the asymmetrical paced group separately to evaluate differential effects in the early *vs.* late-activated EHT zones. However, we did not find indications for such ‘molecular polarization’ in our model (Supplemental Fig. S7 A, see discussion for further details). Consistent with our previous findings under chronic electrical stimulation, symmetrical pacing compared to either asymmetrical or no pacing increased the alpha-actinin abundance normalized to cell number (Fig. 5A + B) or normalized to total protein content (Supplemental Fig. S7 B and C) as a marker of higher cardiomyocyte density or increased sarcomeric mass, a finding which could be corroborated in immunohistochemical stainings (Supplemental Fig. S7 D). In line with the reports from paced dogs, CaMKII- and p38MAPK-abundance (normalized to cardiomyocyte mass, *i.e.*, alpha-actinin abundance) was higher under asymmetrical than under symmetrical pacing while – also consistent - pAKT was considerably lower after asymmetrical pacing (Fig. 5 A + C). In both pacing setups the pCaMKII/CaMKII-ratio was elevated, indicating higher CaMKII-activity which is likely explained by a higher oxidative stress under pacing (Supplemental Fig. S7 E). Cleaved caspase-3 indicating high apoptotic activity could not be detected in any of the EHT groups (Supplemental Fig. S7 F).

## 4 Discussion

In this study, based on the previously established EHT technology, we aimed to develop an *in vitro* model of electromechanical dyssynchrony, motivated by the limited capacity to gain insight on this phenomenon using *in vivo* models only. To our knowledge, this is the first description of a setting that allows for long-term pacing experiments mimicking the electrical aspects of electromechanical dyssynchrony *vs.* synchrony *in vitro*. Combined with quantitative force measurement and afterload stimulus manipulation, our new model potentially provides a robust new tool to gain insight into the biology of electrical dyssynchrony and CRT with important implications as discussed below.

*In vivo* animal models have provided fundamental knowledge into the biology of dyssynchrony and CRT [3]. However, the inability for precise control at the molecular and cellular levels hampers the amount of mechanistic information that can be gained from these models. In this regard, incorporation of cardiac tissues within an *in vitro* model could enable mechanistic studies of dyssynchrony and CRT that can complement the knowledge gained from *in vivo* models. The EHT model involves the combination of viable human cardiomyocytes with a bioartificial support to develop a 3-dimensional construct that recapitulates some of the structural, mechanical, and functional properties of native heart tissue and is amenable to various manipulations (*e.g.*, genetic or pharmacological) to study the effects of biological factors [21].

EHTs develop spontaneous beating in which, in the absence of a specific conduction system, depolarization spreads throughout its 3-dimensional structure from myocyte to myocyte at varying rates and with some irregularity over time. Thus, it is likely that a certain degree of electromechanical dyssynchrony exists in these structures. Recent studies reported that electrical field stimulation considerably improved maturation of both rat and human EHTs [12,[22], [23], [24]]. This was reflected by higher cardiomyocyte density in the center of the tissue constructs, increased connexin-43 abundance, improved sarcomere ultrastructure including regular M-bands, ameliorated cardiomyocyte longitudinal orientation and a higher cytoplasm-to nucleus ratio following chronic stimulation of at least 14 days [12]. All experiments were performed with pacing units that provided uniform excitation of the EHTs, which mimics synchronized conduction to a great extent. Moreover, Nunes et al. [22] showed that a gradual increase in pacing frequency from 1 to 6 Hz over 1 week can further enhance the structure and electrophysiological function of EHTs compared with a low-frequency ramp-up regime from 1 to 3 Hz. Overall, the positive effects of pacing might be the result of electrical stimulation *per se* [25], increased beating frequency or enhanced electromechanical synchrony.

In this context, the asymmetric pacing units that we designed and introduced in the current study enable direct assessment of the effect of electrical synchrony as a single factor. Due to the linear nature of the EHT, under the proper settings these units created longitudinal conduction from the pacing site (early-activated tissue) to the remote end (late-activated tissue) causing a sequence of events which recapitulates the basic electrical components of *in vivo* LV dyssynchrony. On the same logic, uniform field stimulation caused an effect similar to full synchrony in a heart with normal conduction or alternatively, to CRT in a heart with abnormal conduction [3]. Importantly, our ability to demonstrate electrical wave propagation during asymmetric pacing as well as the improved contractile function of the EHT during acute symmetric pacing *vs.* acute asymmetric pacing (Fig. S3 B) are remarkably consistent with the acute effects of dyssynchrony and resynchronization observed on the whole heart level in humans and animal models [6,26], supporting the validity of this model.

As previously reported [12], symmetrically paced low-force EHTs developed more than 2-fold higher forces than non-stimulated EHTs after 16–18 days of continuous pacing. Though less pronounced, this beneficial effect was also visible in EHTs at optimized passive load and high force. The stronger effect in weaker EHTs suggests similarity with findings in man where resynchronization therapy seems the be more effective in hearts with lower ejection fraction [27]. This remarkable effect of pacing was abolished when asymmetric pacing was applied, indicating that enhanced electrical synchrony is a dominant factor leading to the beneficial effects of pacing in this experimental setting. The mechanisms underlying the improved function of symmetrically paced EHTs likely involve both quantitative and qualitative alterations — recruitment of more cardiomyocytes participating in the coordinated contraction of the EHTs and improved function of the single cardiomyocyte as shown previously [12]. Presumably, symmetrical pacing either directly or *via* improved coupling leads to better recruitment of cardiomyocytes in the EHTs, which then contributes to higher force generation. Importantly, in the setting of pathological hypertrophy induced by AE for 7 days [13] the loss of contractile force was markedly accentuated in asymmetrically paced EHTs when compared to symmetrically paced or control EHTs. This result stresses the importance of synchrony in the presence of increased afterload and is consistent with recent clinical findings suggesting a worsened outcome of LBBB in the presence of elevated afterload [28,29].

Consistent with human findings [30], dyssynchrony-associated heart failure (DHF) in the canine pacing tachycardia model resulted in an increase in apoptosis that was suppressed by CRT [20]. One of the most striking changes that was noted in the canine DHF model was a marked decline in AKT phosphorylation which was reversed by CRT. AKT is a pro-survival kinase that reduces apoptosis mediated by AKT–BAD (BCL-2 associated agonist of cell death) signaling. Although apoptosis was not directly detectable in the current study, the fact that AKT phosphorylation was considerably lower after asymmetrical pacing is an important indication for the clinical relevance of our novel *in vitro* model. The relatively low amount of phosphorylated AKT after symmetrical pacing was unexpected and likely reflects a certain electrotoxic effect which however was more than compensated by the beneficial effects of symmetrical pacing. Additional data from the canine model indicated that DHF animals exhibit an increase in p38MAPK and CaMKII activation in the lateral wall of the LV, which were both reversed by CRT. Consistent with that, p38MAPK and CaMKII-abundance was higher under asymmetrical than under symmetrical pacing. However, CaMKII phosphorylation reacted similarly to pacing regardless of the mode, probably reflecting activation by pacing-associated reactive oxygen species. Only symmetrical pacing increased the alpha-actinin abundance, which is considered a marker of higher cardiomyocyte density or increased sarcomeric mass, which was reflected in our histological analysis. The fact that we did not observe biochemical differences between the early-activated and late-activated ends of the EHTs following asymmetric pacing is somewhat inconsistent with the *in vivo* models in which such ‘molecular polarization’ could be noted [10,20]. It is conceivable that the rather short length of the currently used EHTs did not allow for a strong enough level of ‘molecular polarization’, which is in line with our inability to detect mechanical dyssynchrony in our model. Thus, we may suggest that elongation of the EHTs in future studies might provide clear differentiations between the molecular/biochemical findings in both ends and might also enhance the electromechanical findings that were demonstrated in the current study.

While the simplicity of our study is advantageous it might still come with several limitations: Firstly, a relatively short pacing period might not have been sufficient to evolve the full phenotype of dyssynchrony remodeling. Moreover, only effects mediated by and affecting cardiomyocytes can be modeled, leaving out other aspects such as fibrosis, inflammation and neurohumoral processes. The concept of synchronous contraction of the heart, which depends on complex regional anatomy and electrophysiology, can only be partially modeled in a homogenous 8 mm long EHT strip at subphysiological load and resistance conditions [31]. This especially applies as mechanical dyssynchrony, which is depending on resistance and inertia, could not be modeled here. Lastly, molecular phenotyping is partially limited due to the well-known immature state of hiPSC-derived cardiomyocytes, also reflected by the contractile forces of EHT which are approximately one order of magnitude below forces of native human trabeculae.

In conclusion, we demonstrated that asymmetric pacing can recapitulate some important characteristics of *in vivo* cardiac dyssynchrony, especially with regard to electrical dyssynchrony and acute contractile function of the EHT. There are several intriguing implications of our findings. For example, the model can be used to examine whether the biological effects of dyssynchrony might be different in EHTs derived from hiPSCs from CRT responders *vs.* non-responders. In addition, the model can be utilized to learn about possible effects of dyssynchrony on circulating extracellular microRNAs released from the cardiac tissue, as previously suggested [32]. Thus, by using this engineered model, relevant data may be produced to unravel the biological effects of electromechanical dyssynchrony and potentially provide compelling insights into the molecular underpinnings of CRT.

## Disclosures

TE and MNH are co-founders of EHT Technologies GmbH.

## Sources of funding

This work was supported by the German Israeli Science Foundation (GIF), grant no. I-1300-201.14/2015 (TE, MNH and YE), the ERC-AG IndivuHeart (TE), grant no. NCT02417311 and TRAIN-HEART/EU-Marie Skłodowska-Curie Innovative Training Networks (TE and MNH), grant no. 813716.

## Appendix A Supplementary data

Supplementary material Image 1
