# Supplementary material for: Recapitulation of dyssynchrony-associated contractile impairment in asymmetrically paced engineered heart tissue
Source: J Mol Cell Cardiol. 2022 Feb;163:97–105. doi: 10.1016/j.yjmcc.2021.10.001 (PMC8828044; doi:10.1016/j.yjmcc.2021.10.001)
Supplement: Supplementary file 1 — Supplementary material [file mmc1.pdf]

SUPPLEMENTAL MATERIAL

Recapitulation of dyssynchrony-associated contractile impairment in asymmetrically paced engineered heart tissue.

Justus Stenzig[1,2], Marc D. Lemoine[1,2,3], Aaltje M. S. Stoter[1,2], Kinga M. Wrona[1,2], Marta Lemme[1,2], Wesam Mulla[4], Yoram Etzion[4], Thomas Eschenhagen[1,2], Marc N. Hirt[1,2]

[1]Department of Experimental Pharmacology and Toxicology, University Medical Center Hamburg-Eppendorf, Hamburg, Germany; [2]DZHK (German Centre for Cardiovascular Research), partner site Hamburg/Kiel/Lübeck, Germany; [3]University Heart and Vascular Center, University Medical Center Hamburg-Eppendorf, Hamburg, Germany; [4]Department of Physiology and Cell Biology, Regenerative Medicine & Stem Cell Research Center, Ben-Gurion University of the Negev, Beer-Sheva, Israel

Corresponding authors:

Justus Stenzig and Marc N. Hirt, Department of Experimental Pharmacology and Toxicology, University Medical Center Hamburg-Eppendorf, Martinistraße 52, 20246 Hamburg, Germany, Tel: +49-40-7410-52180, Fax: +49-40-7410-54876; E-mail: j.stenzig@uke.de or m.hirt@uke.de

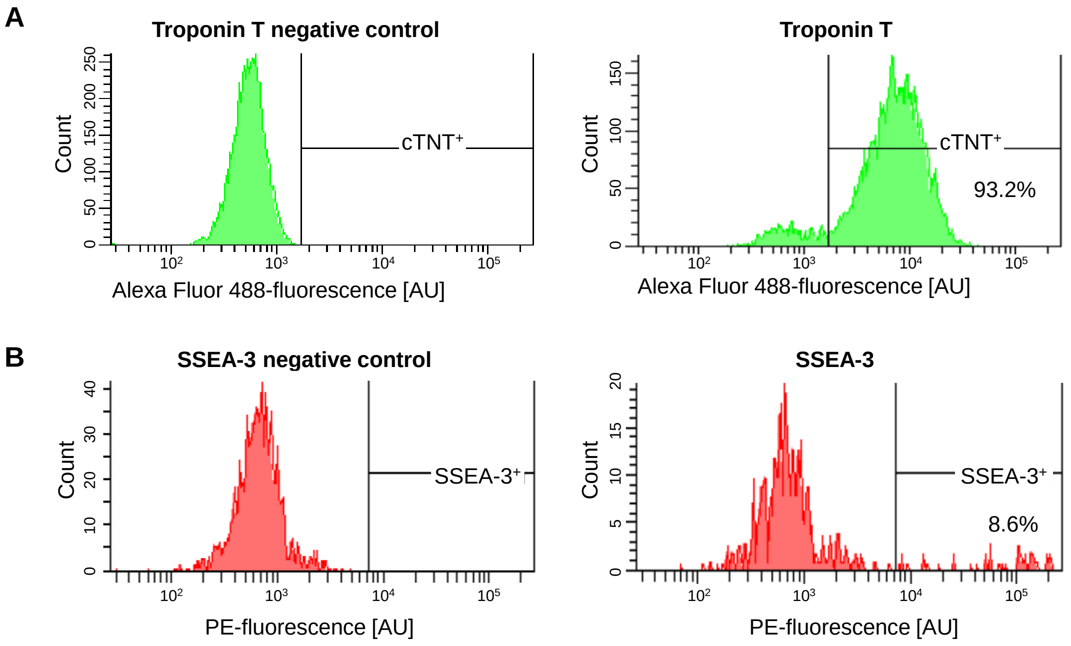

Supplemental Fig. S1: Flow cytometry of cells after cardiac differentiation. Plots show typical values, cells from this batch have been used for the batch included in Fig. 3 C, Fig. 5 B, Supplemental Fig. S2, Supplemental Fig. S4, Supplemental Fig. S5 and Supplemental Fig. S7 B+C. **A** Quantification of cardiac troponin T expressing cells (differentiated cardiomyocytes). Left panel isotype control, right panel troponin T staining. **B** Quantification of SSEA-3 expressing cells (undifferentiated cells). Left panel isotype control, right panel SSEA-3 staining.

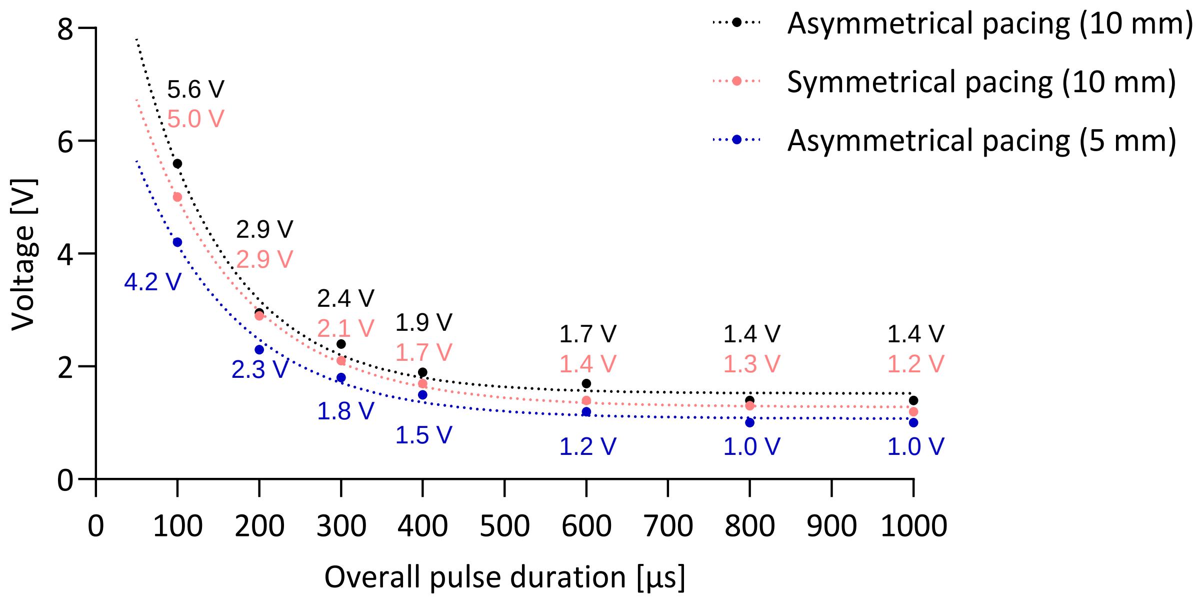

Supplemental Fig. S2: Chronaxie and rheobase. Voltage-duration relationship for the symmetrical and the asymmetrical setup, the latter with 5 and 10 mm inner distance of electrodes. The voltages values represent the threshold at which at least 3 of 6 human EHTs (hEHTs) were paced. In all conditions, maximally 0.2 V above this threshold all EHTs were paced. Rheobase and chronaxie data were R [asymmetrical pacing 10 mm] ≈ 1.4 V, R [symmetrical pacing 10 mm] ≈1.2 V, R [asymmetrical pacing 5 mm] ≈ 1.0 V and C [asymmetrical pacing 10 mm] ≈ 220 ms, C [symmetrical pacing 10 mm] ≈ 260 ms, C [asymmetrical pacing 5 mm] ≈ 240 ms. For all further experiments in this study asymmetrical pacing was performed with the narrow inner electrode distance.

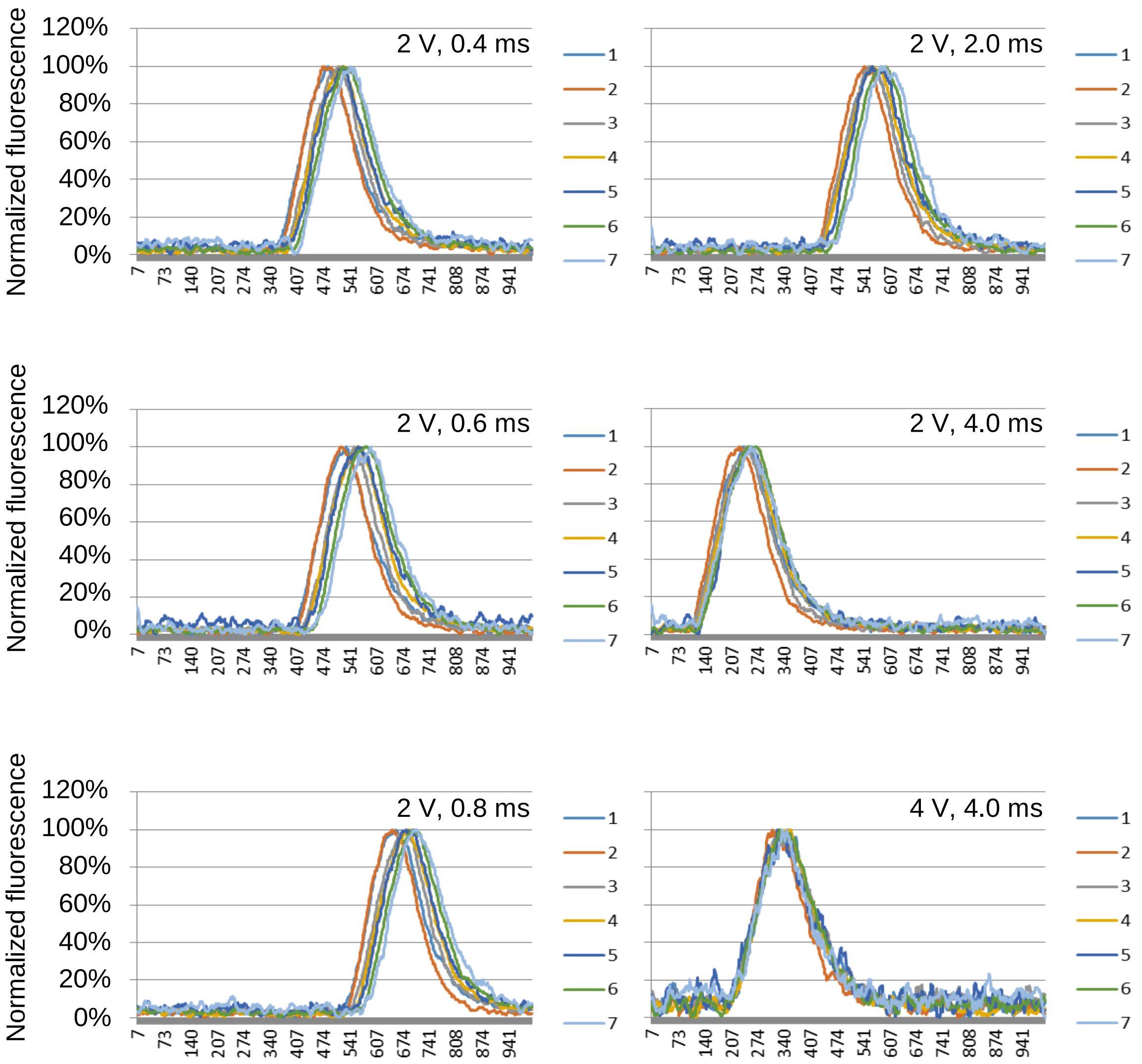

Supplemental Fig. S3: Establishment of asymmetric conditions. Analysis of the Ca^{2+}-wave for the asymmetrical pacing under different stimulator settings. Averaged fluorescence intensities at different locations plotted over time (1 closest to pacing electrodes, 7 farthest to pacing electrodes).

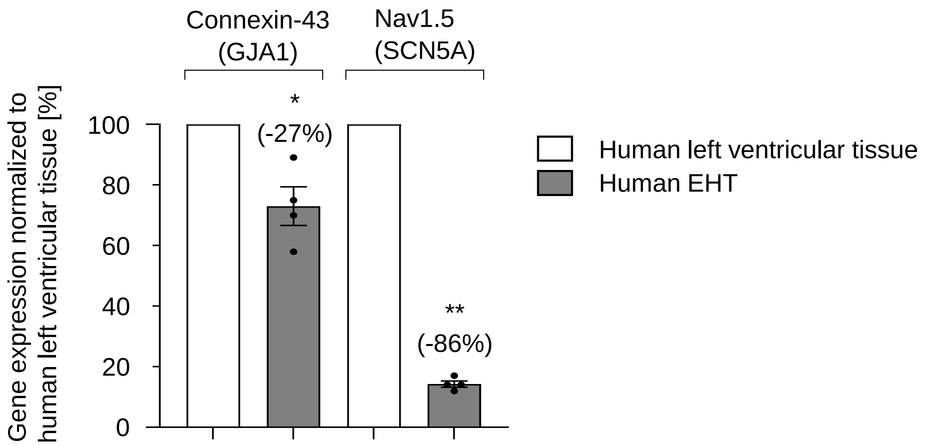

Supplemental Fig. S4: Expression of Connexin-43 and Nav1.5. Relative gene expression of Connexin-43 and Nav1.5 in hEHTs (n = 4) measured by standard quantitative PCR and normalized to a sample of healthy left ventricular cardiac tissue. The ΔΔCt-method was employed, the geometric means of the Ct-values of Glucuronidase beta (GUSB) mRNA and 18S rRNA served as reference (primer sequences: GJA1 forward AGGAGTTCAATCACTTGGCGT, GJA1 reverse CCCTCCAGCAGTTGAGTAGG, SCN5A forward CCCAGGGTGCGGTGAG, SCN5A reverse CGCCGGACACAGCCTTC, GUSB forward ACGATTGCAGGGTTTCACCA, GUSB reverse CACTCTCGTCGGTGACTGTT, 18S forward ATACATGCCGACGGGCGCTG, 18S reverse TTCGAATGGGTCGTCGCCGC. Calculated were parametric one sample tests compared to the control value of 100%.

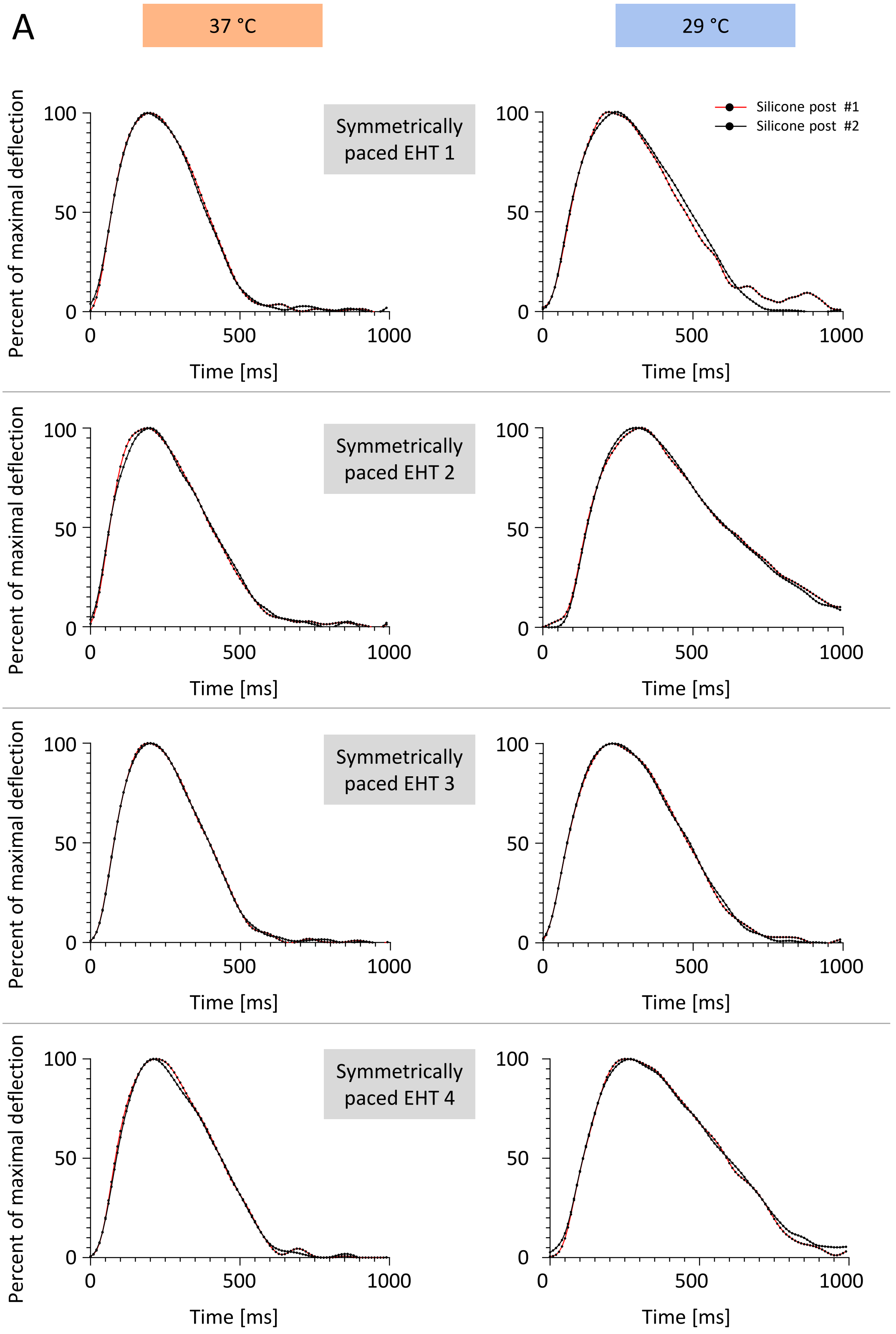

A
37 °C
29 °C
Silicone post #1
Silicone post #2
Symmetrically paced EHT 1
Symmetrically paced EHT 2
Symmetrically paced EHT 3
Symmetrically paced EHT 4
Percent of maximal deflection
Time [ms]
0
50
100
500
1000

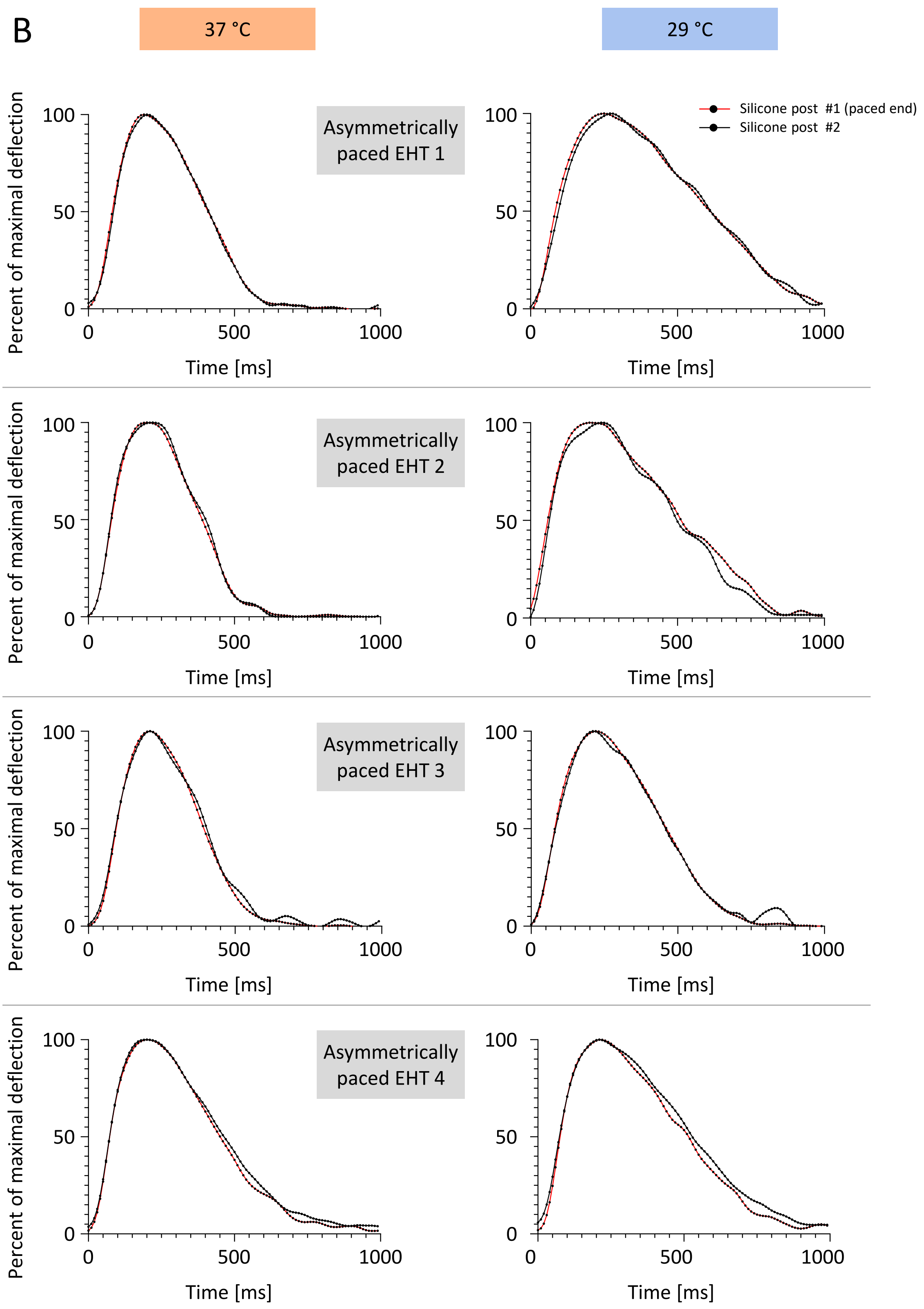

Supplemental Fig. S5: Representative deflection analyses of individual posts of **A** 4 symmetrically paced and **B** 4 asymmetrically paced hEHTs. In B the red curve depicts the deflections of the post which is close to the pacing electrodes. All analyses were conducted under normal cell culture conditions (37 °C) and under hypothermic conditions (29 °C), to slow down kinetics and potentially unmask mechanical dyssynchrony effects.

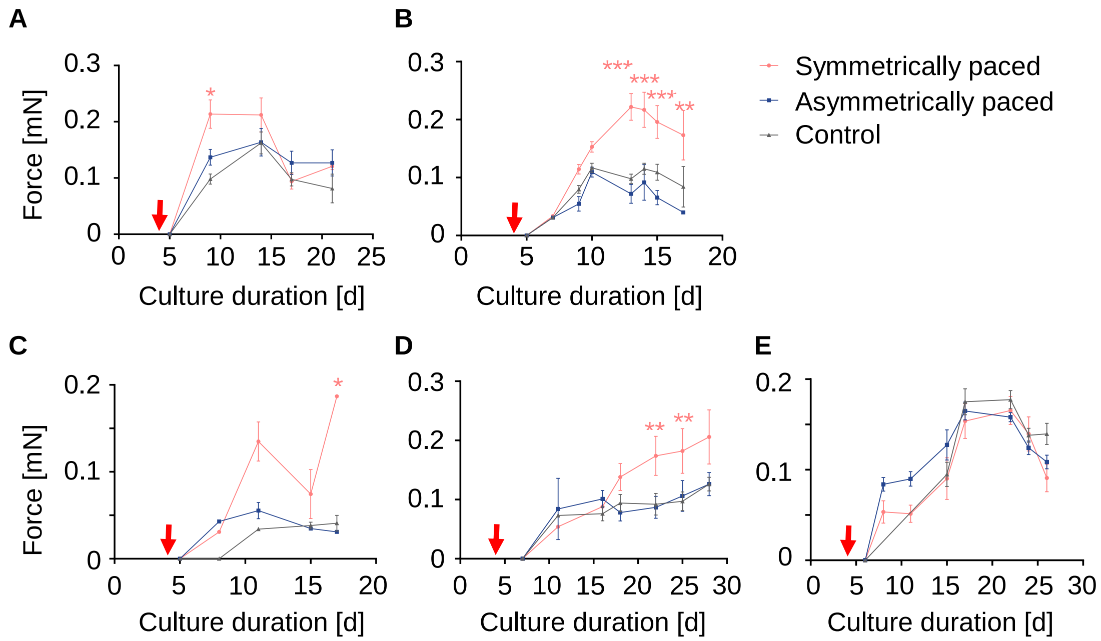

Supplemental Fig. S6: Rat EHT. A-E Force development of 5 series of rat EHTs, all continuously paced from day 4 of culture (red arrow). Two-way ANOVA followed by Sidak's correction for multiple testing. All asterisks denote significant superiority of the symmetrically paced to the non-paced control group. Sample sizes per pacing mode were n = 11-12 [A], n = 11-23 [B], n = 4-8 [C], n = 8-12 [D], and n = 7-9 [E].

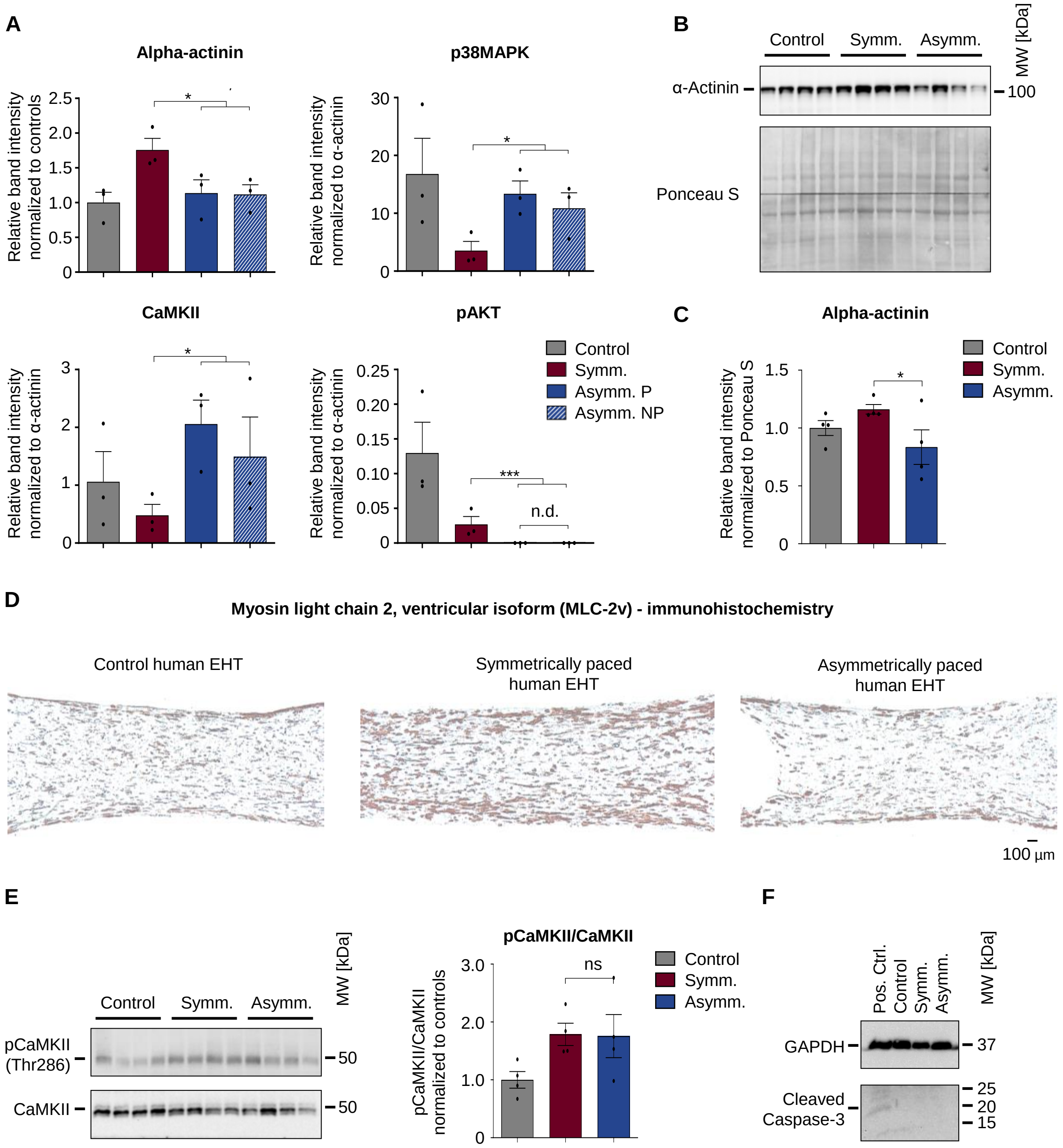

Supplemental Fig. S7: Protein markers of dyssynchrony. A Quantification of Western blot of protein markers of dyssynchrony (p38MAPK, CaMKII and phosphorylated AKT) or a sarcomeric protein (alpha-actinin) at the end of the long-term pacing procedure. In the subset (of all samples from Fig. 5) depicted here the two ends (paced (P) and non-paced (NP) end) of the asymmetrically paced EHTs were analyzed separately. For the alpha-actinin quantification each dot represents protein lysates from ≈ 75,000 input cells. P38MAPK, CaMKII and phosphorylated AKT were all normalized to alpha-actinin values. **B** Western Blot for alpha-actinin and Ponceau S staining in control, symmetrically paced and asymmetrically paced (not separated into different ends) EHTs. **C** Quantification of B. **D** Immunohistochemical staining for myosin light chain 2, ventricular isoform (MLC-2v) in control, symmetrically and asymmetrically paced EHTs. The pacing electrodes for the latter were positioned at the left side. **E** Western Blot for pCaMKII (Thr286) and CaMKII and the respective quantification. **F** Western blot of cleaved caspase 3 as marker of apoptosis in control as well as symmetrically and asymmetrically paced EHTs. HEK cells pretreated with doxorubicin (100 µM) for an hour served as positive and GAPDH as loading control. One-way ANOVA followed by Sidak's correction for multiple testing and n = 3-4 per group in A, C and E.

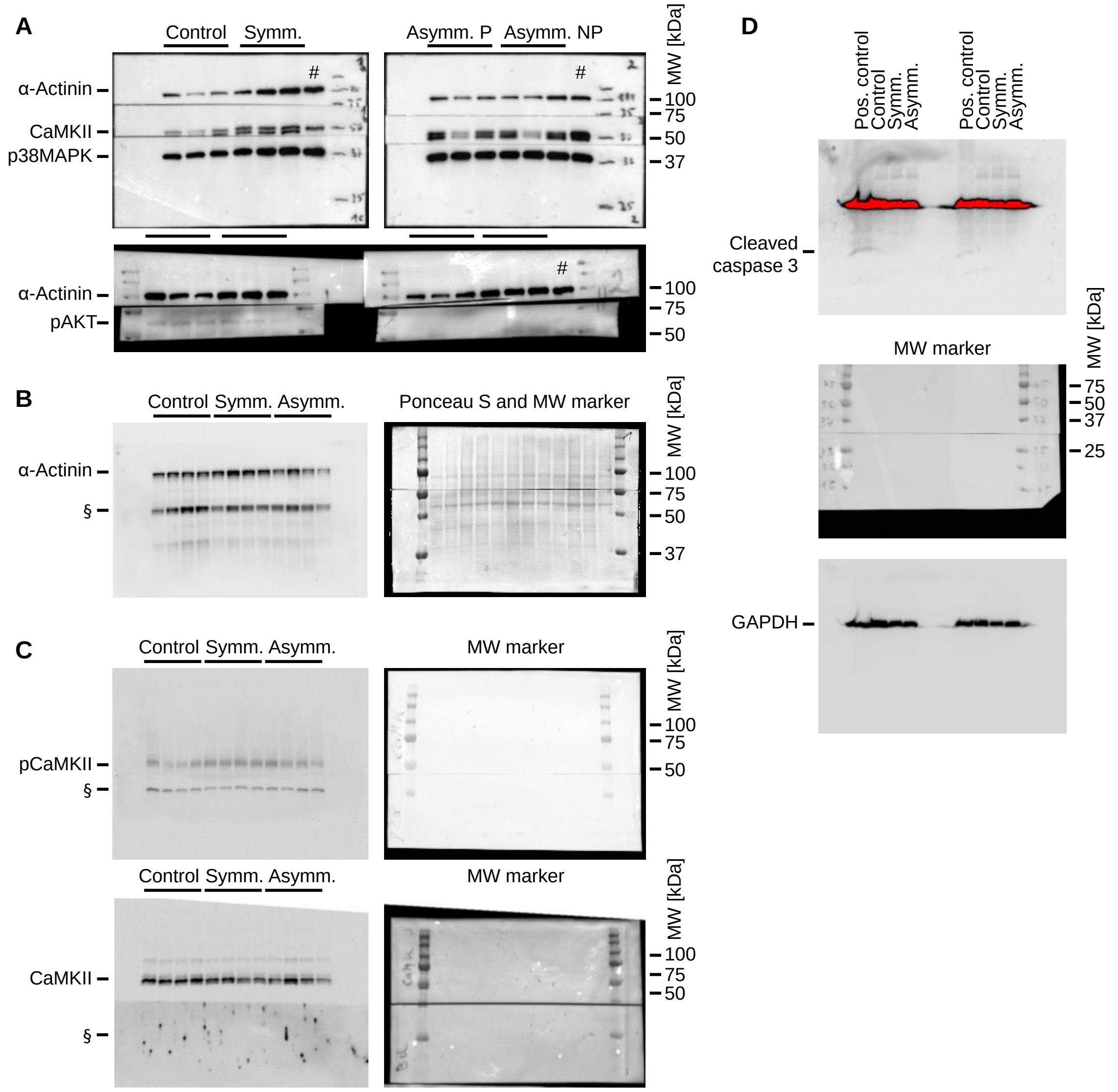

Supplemental Fig. S8: Uncropped Western blot images. A Western blots used in Fig. 5 A-C. **B** Western blots from Supplemental Fig. S7 B. Left panel shows alpha-actinin staining, right panel Ponceau S staining used for normalization. **C** Western blots from Supplemental Fig. S7 E. Left panel shows respective protein staining, right panel molecular weight (MW) markers only. **D** Western blot from Supplemental Fig. S7 F. Top panel and bottom panel show same blot at different exposure time suitable for cleaved caspase 3 (top panel) and for GAPDH (bottom panel). Middle panel molecular weight marker. Right half of blot, same samples, less protein loaded. In all panels: § = further antibody trials, data not shown. # = protein lysate from human iPSC-derived atrial EHT used for staining positive control. Red areas denote overexposed bands.
